# Shear Stress Solutions for Curved Beams: A Structural Analysis Approach

**DOI:** 10.3390/ma17235982

**Published:** 2024-12-06

**Authors:** Renny Guillén-Rujano, Victor Contreras, Argemiro Palencia-Díaz, Wilmer Velilla-Díaz, Adrián Hernández-Pérez

**Affiliations:** 1 Institute of Mechanical Engineering, Universidad Austral de Chile, Valdivia 5110566, Chile; 2 Institute of Naval Architecture and Ocean Engineering, Universidad Austral de Chile, Valdivia 5110566, Chile; victor.palma@uach.cl; 3 Department of Mechanical Engineering, Universidad Tecnológica de Bolivar, Cartagena 130001, Colombia; 4 Department of Mechanical Engineering, Universidad de La Serena, La Serena 1720170, Chile; wilmer.velilla@userena.cl; 5 Department of Earth Sciences, Tecnológico Nacional de México/IT Mérida, Av. Tecnológico km 4.5, S/N, Mérida 97118, CP, Mexico; hepadrian@gmail.com

**Keywords:** curved beams, straight beams, shear stress, mechanics of materials, theory of elasticity

## Abstract

The shear stress on isotropic curved beams with compact sections and variable thickness is investigated. Two new solutions, based on Cook’s proposal and the mechanics of materials approach, were developed and validated using computational finite element models (FEM) for four typical cross-sections (rectangular, circular, elliptical, and triangular) used in civil and mechanical structures, constituting a novel approach to predicting shear stresses in curved beams. They predict better results than other reported equations, are simpler and easier for engineers to use quickly, and join the group of equations found using the theory of elasticity, thereby expanding the field of knowledge. The results reveal that both equations are suitable to predict the shear stress on a curved beam with outer/inner radii ratios in the interval 1<b/a ≤ 5 aspect ratios. There is a maximum relative difference between the present solutions and finite element models of 8% within 1<b/a ≤ 2, and a maximum of 16% in 2<b/a ≤ 5. Additionally, the neutral axis of the curved beam can be located with the proposed solution and its position matches with that predicted by FEM. The displacement at the top face of the end of the curved beam induces a difference in the shear stress results of 8.0%, 7.0%, 6.5%, and 2.9%, for the circular, rectangular, elliptical, and triangular cross-sections, respectively, when a 3D FEM solution is considered. For small b/a ratios (near 1), the present solutions can be reduced to Collignon’s formula.

## 1. Introduction

Structural beams have been studied for a long time; straight beams, beams on elastic foundations, and curved beams (CBs) are still an important topic of study in mechanical and civil engineering [1,2,3,4,5,6,7,8,9,10,11,12,13], as well as in related fields such as architecture [14,15,16], biomechanics [17,18,19], and electronics [20,21]. Enormous structures such as bridges, viaducts, and buildings with innovative and aesthetically pleasing shapes [16,22], vehicles, aircraft, spaceships with high performances [23], and small structures such as biomedical devices and tools, prosthetics [24,25], and also electronic parts [20] are developed by using the available knowledge on the mechanical behavior of CBs. Much research has been conducted to find solutions for predicting the stress, strain, and deformation of various types of beams. Specifically, for CBs, solutions for predicting the circumferential, radial, and shear stresses are available for rectangular cross-sections of uniform thickness since Timoshenko and Goodier [26] developed it using the theory of elasticity in 1934. Later, in 1960, Eason [27] proposed using the elastic–plastic bending of a compressible curved bar; in 1968, Lekhnitskii [28,29] employed the linear elasticity anisotropic theory, and in 1993, C. Bagci [30] solved the differential equation in polar coordinates; however, all of these methods lead to Timoshenko’s solution. Prior to Timoshenko’s solution, Winkler addressed the curved beam problem as an extension of its own elementary theory of straight beams developing an approximate formula for predicting the circumferential stress, which today is known as Wrinkler’s equation [31,32,33]; nevertheless, large errors are predicted due to the considerations of the centroidal axis as the neutral axis and neglect of curvature [31]. Some improvements were reached when an alternative form of Wrinkle’s equation was introduced by himself, resulting in being applicable to almost all types of sections, but not all, according to Bleich [34] and Anderson [35], who established that the cross-sections of curved members tend to distort under load, resulting in greater stresses than those predicted by Wrinkler’s theory. Cook [36] introduced three alternative solutions to Wrinkler’s theory to improve accuracy on circumferential stress. Later, C. Bagci developed an interesting solution for predicting not only the circumferential stress, but also the radial and shear stresses for CB with exponential and T-shaped cross-sections [30]. Cook’s equations apply for most cross-sections [36,37], but errors are obtained in those CBs having flanges, and for such cases, Bleich’s correction factors must be used. The first general formula for the computation of the shear stresses in CBs was published by Wang in 1967 [38], the second by Birger in 1968 [39], the third by Oden in 1981 [40], and two more solutions by Liu in 1985 [41] and Yu in 2005 [42]. Despite the large investigation reported in the literature, a general equation to predict the shear stress in a CB with a symmetric general cross-section is an incomplete work to date, existing only the 2D exact elasticity solutions for a CB with constant thickness developed by Timoshenko, as was mentioned before; the exact solution for exponential thickness and T-shaped section, developed by C. Bagci in 1993 [30]; and the approximate equations using mechanics of materials concepts presented by Wang, Birger, Oden, Liu, and Yu. The formulas of Wang and Birger turned out to be the same equations after an algebraic manipulation, as well as those of Liu and Yu, which, despite having different structures, yield the same results. Another precise, but complex, theoretical study of CBs was developed recently by Iandiorio in 2022 [43]. This solution only gives an exact solution for rectangular cross-section, and is strictly applied to double symmetric cross-sections, with the particularity that for other cross-sections different to rectangular ones, the procedure requires the numerical evaluation of some geometric integrals. Many other studies have been conducted to find solutions for CBs; those are commonly numerical procedures as an alternative solution to the theory equations due to the complexity of the involved differential equations [44,45,46,47,48,49,50,51], which are influenced by cross-section geometry [30]. Others are experimental [52], and both are conducted to validate the models. Here, we limit ourselves to mentioning two studies: the first, made by Chung et al. in 1982 [53], which added electric strain gauges to CB aluminum (204-T351) specimens. They made measurements of circumferential and radial stresses and reported good agreement between experiments and the exact elasticity solution; however, an experiment for shear stresses could not be carried out. And the second was made by Hassan in 2014 [54]. He conducts an experimental and analytical study of bending stresses and deflection in composite laminated CB made of epoxy and fiberglass. He found an agreement of 95% among numerical, experiments and theoretical results in deflection. Prasad et al. [55] in 2016 conducted an experimental stress analysis of cross-section CBs using strain gauges added to 6351-T4 aluminum specimens; they measured and compared the circumferential stresses against Wrinkler’s theory, concluding that the results obtained are in close accordance. Ahuett-Garza et al. [56], in 2014, successfully developed two planar-compliant mechanics to test semicircular beams as hinges in large deflection. Yanze et al. [57] conducted a theoretical and experimental analysis of thin-walled curved rectangular box beam under in-plane bending. CB specimens made of Q235 steel were manufactured using a cutting machine and welding procedure; after that, strain gauges were added at the top surface, and with an acquisition data system, the strain was recorded when the load was applied. The experimental results showed small differences with respect to FEM, making this method for measuring deflections a good option for experimental procedures. Other experimental research has been made for detecting deflection [58], and stresses [59] in CB [60]. From the three analytical models proposed by Cook [36] to predict the circumferential stress in a CB, it is possible to obtain three equations for predicting the radial and shear stresses. This research aims to obtain the three shear stress equations and evaluate their precision with respect to the FEM simulation for typical compact cross-section. Four typical sections (rectangular, circular, elliptical, and triangular) were analyzed because these are the most used cross-sections in many engineering applications as tools used in cranes for tilting walls, dragging and lifting large loads, rope tying accessories for rescue operations or extreme sports, for design of thin-wall cross sections, for unidirectional composite materials, among other applications [61,62].

## 2. Materials and Methods

### 2.1. Shear Stress Equations Using a Mechanics of Materials Approach

In order to develop a solution for CBs, it is assumed that the material is homogeneous, isotropic, exhibits an elastic behavior, the strain and rotations are small, the plane sections before loading remain plane after loading, the state of stress is dominantly one-dimensional, and the applied loads lie in the plane of symmetric [61].

**Figure 1:**
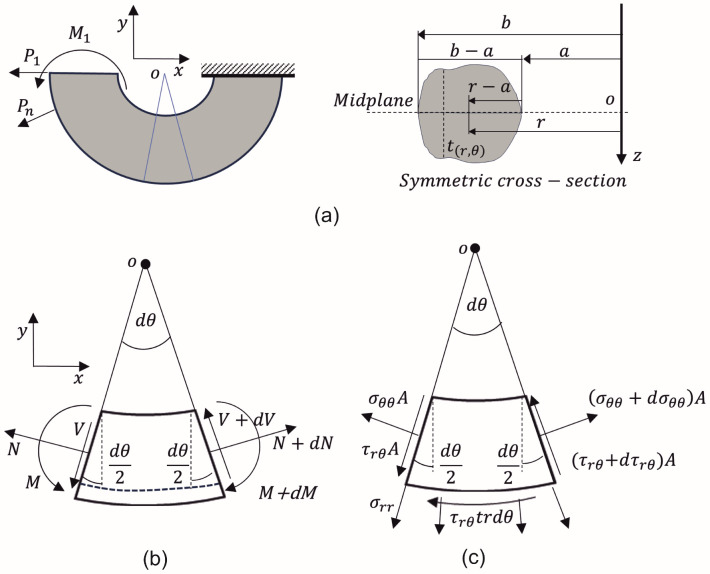
Schematic of a CB: (**a**) with a symmetric general cross-section, (**b**) differential element as a function of internal loads, (**c**) differential element as a function of stresses (adapted from [61]).

Figure 1a shows a CB schematic with a symmetric general cross-section, where $P_{1},P_{n},M_{1}$, represent the external applied loads. A small section from this CB is taken to represent the internal loads acting on the beam (Figure 1b). In this figure, *V* is the internal shear load, *M* is the internal bending moment, *N* is the internal normal load, and θ is the angle of the CB. The small section in Figure 1b is split into two parts (separated by dashed lines), and the superior part is shown in Figure 1c to represent the shear stress $\tau_{r\theta }$. It is important to point out that the thickness *t* depends only on the radius *r*, because the cross-section remains constant throughout the entire θ, i.e, $\left(\tau_{\left(r,\theta \right)}=\tau_{\left(r\right)}\right)$. Figure 1b represents a differential portion of the CB shown in Figure 1a. From this figure, taking moments around the center of curvature o, and using the mechanics of materials (MM) theory, an equation for the circumferential stress (Equation (1)) is obtained [61,63]. The first term in this equation represents the normal stress due to axial force *N*, and the second is the normal stress due to bending moment *M*. Equation (1) offers good agreement with the theory of elasticity when N=0 [36,64], but when N≠0, the equation becomes inaccurate. Cook [36] proposed two modifications (Equations (4) and (5)) to improve its accuracy and correct the problems. As the shear stress equation is obtained using the circumferential stress equation, it is important to evaluate its accuracy against FEM results. The radial stress $\left(\sigma_{rr}\right)$ is determined by summing the contributions of radial and perpendicular forces to the CB element (Figure 1c), as depicted by Equation (2). Note that Equation (3) only depends on the geometric variables.

(1) $$\sigma_{\theta \theta }=\frac{N}{A}+\frac{M(A-rA_{m})}{Ar(RA_{m}-A)}$$

(2) $$\sigma_{rr}=\frac{A^{\prime }}{Atr}N+\frac{M(AA_{m}^{\prime }-A^{\prime }A_{m})}{Atr(RA_{m}-A)}$$

(3) $$A=\int_{a}^{b}dA;A_{m}=\int_{a}^{b}\frac{dA}{r};A^{\prime }=\int_{a}^{r}dA;A_{m}^{\prime }=\int_{a}^{r}\frac{dA}{r};R=\frac{1}{A}\int_{a}^{b}rdA$$

where *r* is the radial position, *A* is the cross-section area, *a* is the inner radius, *b* is the outer radius, *t* is the thickness, and *R* is the distance from the center of the curvature to the centroid of the beam cross-section. It is important to point out that Equation (1) becomes Navier’s equation for straight beams (SB) when *R* tends to infinity [61,65].

As was mentioned previously, Cook [36] proposed two modifications (Equations (4) and (5)) based on FEM analysis to improve the accuracy of the circumferential stress. Firstly, for Equation (4), the term N/A of Equation (1) was replaced by $\left(Nr_{n}\right)/\left(Ar\right)$, arriving at Equation (4). Secondly, a more complex consideration was made for Equation (5), where the term N/A was replaced by $\left(N/A\right)/\left(r_{n}/r+r/R-1\right)$, where $r_{n}=\left(1/A\right)\int dA/r$. These equations are used herein to obtain two new expressions for the computation of the shear stress in a CB. Despite these equations being expressed in different ways by Cook, they are the same.

(4) $$\sigma_{\theta \theta }=\frac{N}{rA_{m}}+\frac{M(A-rA_{m})}{Ar(RA_{m}-A)}$$

(5) $$\sigma_{\theta \theta }=\frac{N}{A_{m}}\left(\frac{1}{r}+\frac{\left(A_{m}R-A\right)\left(r-R\right)}{I_{m}}\right)+\frac{M(A-rA_{m})}{Ar(RA_{m}-A)}$$

(6) $$I_{m}=\int_{a}^{b}{\left(R-r\right)}^{2}\;dA$$

By taking moments about the center of curvature o of Figure 1c,

(7) $$-\int_{a}^{r}r\sigma_{\theta \theta }dA+\int_{a}^{r}r\left(\sigma_{\theta \theta }+d\sigma_{\theta \theta }\right)dA-\tau_{r\theta }r^{2}td\theta =0$$

(8) $$\tau_{r\theta }r^{2}t=\int_{a}^{r}r\frac{d\sigma_{\theta \theta }}{d\theta }dA$$

By including Equation (5) into Equation (8),

(9) $$\tau_{r\theta }r^{2}t=\int_{a}^{r}r\frac{d}{d\theta }\left(\frac{N}{A_{m}}\left(\frac{1}{r}+\frac{\left(A_{m}R-A\right)\left(r-R\right)}{I_{m}}\right)+\frac{M(A-rA_{m})}{Ar(RA_{m}-A)}\right)dA$$

and by simplifying and rearranging terms in Equation (9), a new equation to calculate the shear stress in a CB is obtained by Equation (10). Note that *N* and *M* depend on θ, and terms $I^{\prime }$ and *Q* (Equation (11)) are easily calculated.

(10) $$\tau_{r\theta }=\frac{1}{A_{m}r^{2}t}\left(A^{\prime }+\frac{\left(A_{m}R-A\right)\left(I^{\prime }-RQ\right)}{I_{m}}\right)\frac{dN}{d\theta }+\frac{\left(AA^{\prime }-A_{m}Q\right)}{r^{2}tA\left(RA_{m}-A\right)}\frac{dM}{d\theta }$$

where

(11) $$I^{\prime }=\int_{a}^{r}r^{2}dA;Q=\int_{a}^{r}r\;dA$$

**Figure 2:**
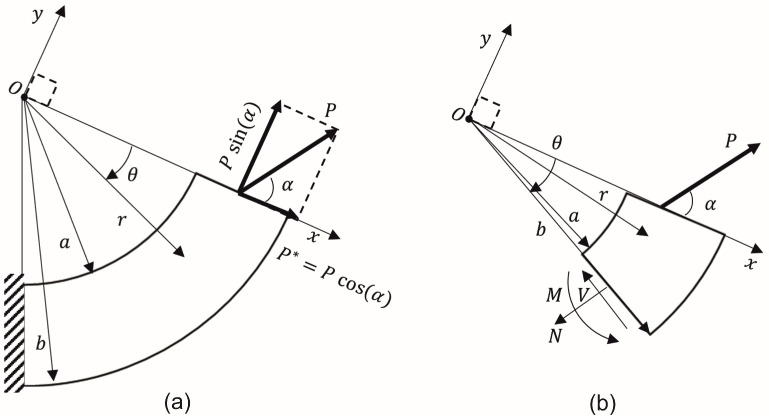
(**a**) CB under an arbitrary concentrated force *P*, (**b**) general free-body diagram of a CB element to represent the internal loads.

To study the shear stress behavior in a CB, a concentrated force (P) is placed at the center of the cross-section, as observed in Figure 2a. *P* can be projected in a tangential (Pcos(α)) and a radial (Psin(α)) directions. To consider the variations in internal loads along the CB path, Figure 2b shows an element at specific θ orientation. By performing an equilibrium analysis on Figure 2b, the internal forces and bending moments become the following.

where

(12) $$\begin{array}{cc} V & =P\cos(\alpha +\theta )\\ N & =P\sin(\alpha +\theta )\\ M & =RP\left(\sin(\alpha +\theta )-\sin(\alpha )\right) \end{array}$$

By including Equation (12) in Equation (10), the shear stress $\tau_{r\theta }$ becomes a function of both α and θ angles.

(13) $$\tau_{r\theta }=\left(\frac{A^{\prime }}{A_{m}}+\frac{\left(A_{m}R-A\right)\left(I^{\prime }-RQ\right)}{I_{m}A_{m}}+\frac{R(AA^{\prime }-A_{m}Q)}{A(RA_{m}-A)}\right)\frac{P}{r^{2}t}\cos\left(\alpha +\theta \right)$$

For α=0, the normal component (Psinα) vanishes, and only the tangential load (Pcosα=P) remains [23,24], i.e, the applied load *P* is parallel to the CB cross-section. If α≠0, the parallel load is $P^{*}=P\cos\alpha$. The MM equation for computing the shear stress in a CB is

(14) $$\tau_{r\theta }=\left(\frac{A^{\prime }}{A_{m}}+\frac{\left(A_{m}R-A\right)\left(I^{\prime }-RQ\right)}{I_{m}A_{m}}+\frac{R(AA^{\prime }-A_{m}Q)}{A(RA_{m}-A)}\right)\frac{P}{r^{2}t}\cos\left(\theta \right)$$

where Pcos(θ) is the shear load at the orientation θ. Equation (14) represents our first MMs solution for the CB problem, and will be referred to as MM_1_. Two additional equations for $\tau_{r\theta }$ can be obtained by separately including Equation (1) into Equation (8), and Equation (4) into Equation (8), i.e, Equations (15) and (16), respectively. Cook [36] has widely discussed the problems and limitations of Equations (1) and (4), as from Equations (15) and (16), the same limitations are obtained, and have been considered here. For comparative purposes, both equations are studied herein. However, these equations might be inaccurate due to the dependency of $\tau_{r\theta }$ to $\sigma_{\theta }$ according to Equation (8).

(15) $$\tau_{r\theta }=\left(\frac{RA^{\prime }-Q}{RA_{m}-A}\right)\frac{P}{r^{2}t}\cos\left(\theta \right)$$

(16) $$\tau_{r\theta }=\left(\frac{A^{\prime }}{A_{m}}+\frac{R(AA^{\prime }-A_{m}Q)}{A(RA_{m}-A)}\right)\frac{P}{r^{2}t}\cos\left(\theta \right)$$

Equations (15) and (16) also represent solutions by using the theory of MM, and will be named MM_2_ and MM_3_, respectively. Equations (14)–(16) are formulated under the assumption that bending moment *M* in Equation (1) yields an uniaxial $\sigma_{\theta }$ and straight cross-sections remain plane under bending. However, Equation (15) can be reduced to Collignon’s formula [66], as shown in Appendix A, which is also used for general cross-sections in SBs. According to Cook, if *N* vanishes, large errors are obtained, and the circumferential stress is underestimated. Equations (4) and (5) were considered herein to obtain Equations (14) and (16), respectively. It is important to note that the first and second terms of Equation (14) represent shear stress approximations induced by the two tangential stress corrections considered by Cook [36]. The first term results from the consideration of ($Nr_{n}$)/(Ar), while the second arises from (N/A)/($r_{n}/r+r/R-1$), both of which are approximations based on Cook’s observations obtained from FEM analysis.

### 2.2. Finite Element Analysis

**Figure 3:**
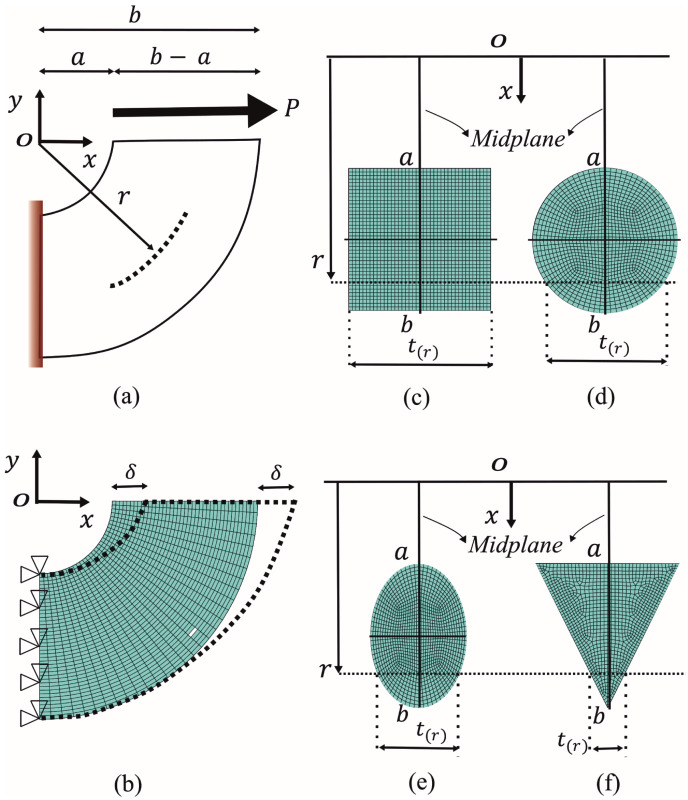
Schematic finite element mesh for the CB specimen. (**a**) model of the CB loaded. (**b**) FEM model with border conditions. (**c**) Mesh of rectangular cross-section. (**d**) mesh of circular cross-section. (**e**) mesh of elliptical cross-section. (**f**) mesh of triangular cross-section.

For a basis of comparison against the proposed solutions, a finite element model (FEM) is developed by using the commercial software ANSYS (2023R1) [67]. To compute shear stress, the brick element SOLID186 and plane element PLANE183 with quadratic interpolation are employed. PLANE183 is defined by eight nodes (four corner nodes and one mid-side node between two corners) having three degrees of freedom at each node (translations along the x,y, and *z* axes). SOLID186 is defined by twenty nodes (eight corner nodes and one node between two corners). Because this element is compatible with the PLANE183 [67], the 3D models of the CB were constructed extruding previously meshed plane areas contained in the xz plane along a circular profile of radius r=a and 90 degrees (see Figure 3). Four typical sections (rectangular, circular, elliptical, and triangular) are studied (see Figure 3c–f) because these are the most used cross-sections in many engineering applications. The simulations were performed considering an isotropic linear elastic material with an elastic modulus (E) of 200 GPa and a Poisson’s ratio (ν) of 0.28, which represents the elastic behavior of the structural steel. Table 1 lists the dimensions and b/a ratios of the CBs analyzed, keeping the depth of the beam as 100 mm (b−a=100 mm). Ratios b/a=3 and b/a=2 with the rectangular cross-section are compared against the solution presented by Timoshenko and Goodier [26]. For all models, a maximum thickness of 100 mm was considered. To simulate the application of the tangential load *P* at the top face of the CB (see Figure 3a), an uniform displacement of δ=0.1 mm was induced on the same face (see Figure 3b). Nodes at the lower face (yz plane) were fixed along the x,y and *z* axes (u,v,w=0) in order to simulate fixed support and avoid the free-body motion of the CB. An analysis of convergence was carried out to investigate the effect of meshing size on $\tau_{r\theta }$, which turned out negligible (variation of less than 0.37%). For this purpose, elements of a nominal size ranging from 10×10 mm^2^ to 1.25×1.25 mm^2^ were considered at the cross-section of the rectangular CB (other models employed similar sizes), with 15 to 30 divisions equally spaced, and 6.0 to 3.0 degrees along the arc for all cases studied. All FEM models with the number of nodes between 52,941 and 410,751 attain the same shear stress values along the midplane, indicating that the FEM is robust and self-consistent. The results of this stringent convergent analysis yielded models consisting of 49,600 elements with a nominal size of 2.5×2.5 mm^2^, having 20 divisions equally spaced at the cross-section and 4.5 degrees along the arc for the CB with a rectangular cross-section. The same mesh configuration was used for parametric analysis; only the ratio b/a was changed.

## 3. Results and Discussion

### 3.1. Validation of the Shear Stress Equations

**Figure 4:**
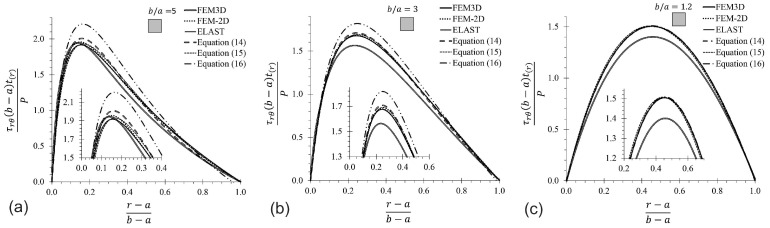
Shear stress comparison for CBs with rectangular cross-section (for midplane axis at θ=0°). (**a**) b/a=5, (**b**) b/a=3, (**c**) b/a=1.2.

Prior to the present a parametric analysis, the proposed MM solutions for $\tau_{r\theta }$ (Equations (14)–(16)) were validated by comparing such solutions to FEM and elasticity [26], as shown in Figure 4. This was conducted only for a CB with a rectangular cross-section and an aspect of ratio b/a= 5, 3, and 1.2. In this figure, the variation in the normalized shear stress, divided by the average shearing stress (P/(t(b−a))), located at the midplane along the beam depth a≤r≤b for θ=0°, is plotted versus the normalized radial position ((r−a)/(b−a)) along the beam depth. A close-up view near the peak of the curve is provided for each figure to visualize the differences among the solutions. As appreciated in the three charts, the curves describe the same behavior, predicting almost the same values for the interval 0≤(r−a)/(b−a)≤0.17, and the 2D-elasticity and 2D-FEM solutions converge to the same results at the whole interval. The 3D-FEM solution underestimates a maximum value, with a 7.0% of difference with respect to the 2D-elasticity; this is attributed to the boundary condition of displacement (Figure 3b), which is related to the Poisson’s ratio and affects the shear stress. Nevertheless, Equations (14) and (15) are in agreement with the theory of elasticity, while Equation (16) overestimates the maximum shear stress in 12%, for a ratio of b/a=5. This difference suggests that Equations (14) and (15) are more accurate than Equation (16). The maximum difference between the 3D-FEM and MM solutions for the shear stress is 14%, but it was noticed that the deviation of neutral axes with respect to FEM is of 0.02·(r−a)/(b−a). It is also observed in Figure 4 that the shear stress $\tau_{r\theta }$ vanishes on inner and outer surfaces at r=a and r=b, and increases rapidly from r=a to a maximum value located at the neutral axis near to the r=a side when the b/a ratio grows. One last observation is that as the b/a ratio decreases, the shear stress curve becomes more symmetric; i.e., the neutral axis comes near the centroidal axis.

### 3.2. Poisson’s Ratio Sensibility in CB

**Figure 5:**
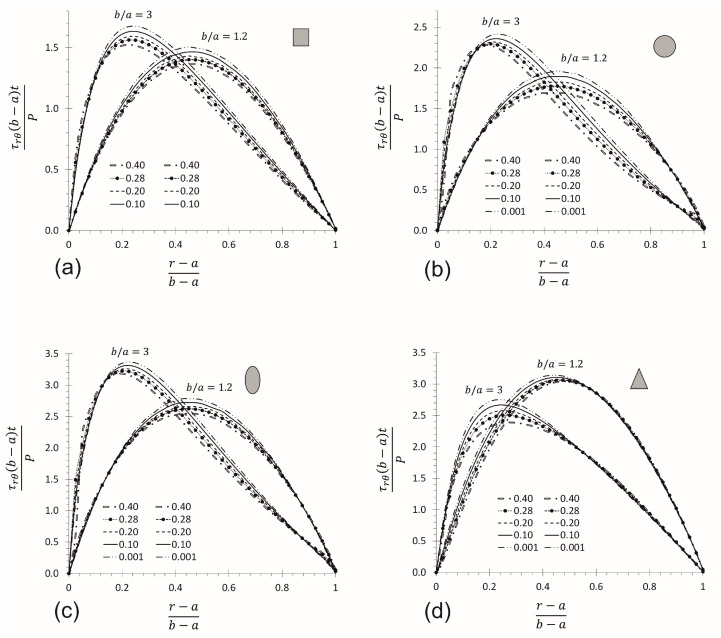
Poisson’s ratio effect by using FEM on CBs with ratios of b/a= 3 and 1.2 and different cross-section shape: (**a**) rectangular, (**b**) circular, (**c**) elliptical, and (**d**) triangular cross-section (for a midplane axis at θ=0°).

For predictions of the shear stress in a CB on regions near the midplane, the 2D-elasticity equation developed by Timoshenko and Goodier [26] can be considered a good approximation for a 3D analysis, because its difference is only 7.0% (as previously mentioned), despite the difference increases for predictions far from the midplane. To validate the application of the MM equations, it was necessary to simulate four CB models in FEM with different cross-sections (rectangular, circular, elliptical, and triangular). It is well known that stresses are independent of the Poisson’s ratio; nevertheless, five Poisson’s ratios were considered for the rectangular cross-section (ν= 0.4, 0.28, 0.20, 0.1, and 0.001) with ratios of b/a= 3 and 1.2 due to the border condition of displacement given at the end of the CB. Figure 5 shows results in the interval a≤r≤b for θ=0° at the midplane of the CBs. The border condition of displacement given at the end of the CB produces a slight difference in the results when Poisson’s ratio is considered. The maximum difference was found for a CB with a circular cross-section (8.0%), followed by the rectangular, elliptical, and triangular cross-sections, with 7.0%, 6.5%, and 2.9%, respectively. This small difference is considered negligible. It is important to note that Figure 5d (triangular CB) describes different behavior on the right side of the curve, compared to Figure 5a–c. This remarkable difference is because the rectangular, circular, and elliptical CB have two perpendicular symmetric axes in their cross-sections, but it is not the same in the triangular CB. The neutral axis location is strongly influenced by the symmetric condition of the cross-section, but not by the Poisson’s ratio. CBs with rectangular, circular, and elliptical cross-sections tend to exhibit a symmetrical stress profile as the b/a ratio decreases, and the neutral axis for all shear stress profiles, including the triangular profile, tends to be located near the centroid of the cross-section.

### 3.3. CB vs. SB for Different Cross-Section

**Figure 6:**
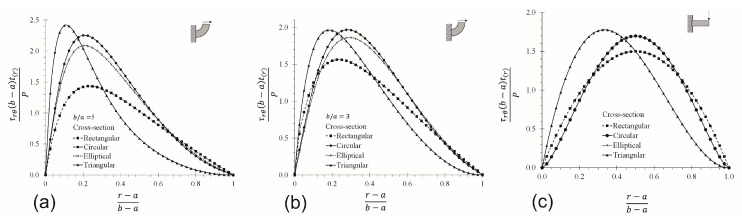
Shear stress at midplane axis and θ=0° by using FEM. (**a**) CBs: b/a=5, (**b**) CBs: b/a=3, (**c**) SBs.

The effects of normalized shear $\tau_{r\theta }$ stress on CBs with ratios of b/a= 5 and 3, and on SB (using the Collignon’s formula), are studied in Figure 6. By comparing Figure 6a,b, it is observed that the b/a ratio has a strong influence on the shear stress behavior. The peak values are greater on CBs than SBs for the cross-section with two symmetrical axes, but are not the same for the triangular section, which depends on the location of the section concerning the radio of curvature, i.e, the stress profile is different when the base of the triangular cross-section is located at r=b than when is located at r=a. The shear stress in SB (Figure 6c) seems to describe a parabolic symmetric behavior for all cases shown except for the triangular cross-section. This parabolic trend is demonstrated for SB with a rectangular cross-section [66], and occurs because the neutral axis coincides with the centroid (r=(b+a)/2) and has a symmetric axis normal to *r*. For a triangular cross-section, these points are located at r=2(b+a)/3, and do not have a symmetric axis with respect to *r*. CBs with the largest b/a ratio tend to reach the shear stress peaks far from the centroid (Figure 6a,b), and their values are larger than those predicted by Collignon’s formula, increasing as b/a grows. This indicates that latter is incorrect in those cases.

### 3.4. Parametric Analysis of CB

**Figure 7:**
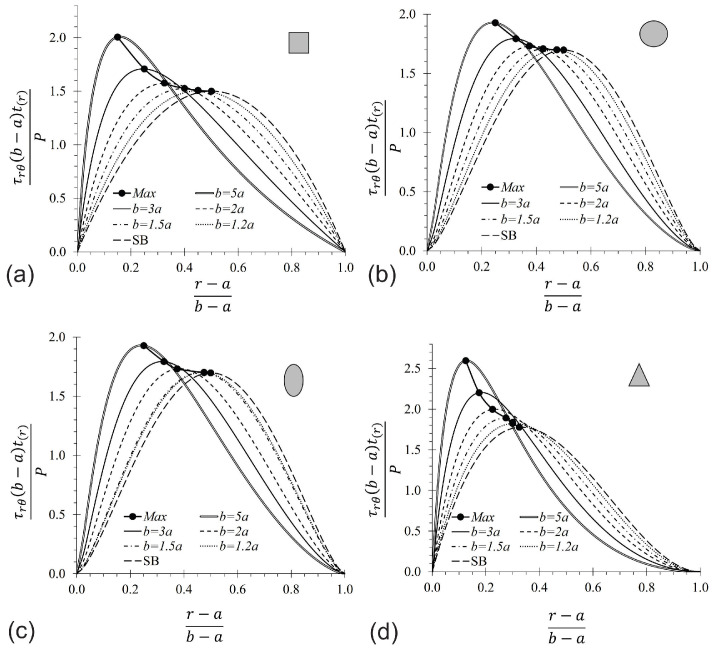
Parametrical study of the CBs by using Equation (14). (**a**) Rectangular, (**b**) circular, (**c**) elliptical, and (**d**) triangular cross-section (for midplane axis at θ=0°).

A parametric analysis was conducted in order to study the influence of the aspect of ratio b/a on the shear stress. Six b/a ratios were considered: b/a= 5, 3, 2, 1.5, 1.25, and 1.2. The results are shown in Figure 7, Table 2 and Table 3. For each Figure 7, the shear stress peak is located at the neutral axis, which is highlighted with a black dot and connected with a black line. In the four cases represented in Figure 7, it is observed that the shear stress decreases and the neutral axis tends to be closer to the centroid of the cross-section when the b/a ratio tends to the unity. It is also observed that when the b/a ratio is close to unity, Collignon’s formula predicts values almost equal to FEM. We note the close agreement among the results for b/a= 1.2 and SB. In conclusion, for b/a≤1.5 Collignon’s formula can be used with a small error to predict the shear stress in CB, but for 1.5<b/a≤5, it is recommended to use a CB solution. In fact, the CB solution could be used in the whole range 1<b/a≤5. Table 2 lists the neutral axis location of the CBs analyzed by FEM. Values identified with (*) represent the solution obtained by Timoshenko and Goodier [26] for a CB with a rectangular cross-section. It is observed that the error between the FEM and the elasticity solutions is less than 2.5%. The neutral axis can be located by Equations (15) and (16); the deviation is almost imperceptible, with an absolute difference of less than 0.03·(r−a)/(b−a) among both equations, and predicts similar values than FEM and elasticity solution, even for larger b/a ratios. Additionally, it can be seen in Figure 7 and Table 2 that the coordinate of the neutral axis is closer to the centroid ((r−a)/(b−a)=0.5) as the b/a ratio decreases.

**Figure 8:**
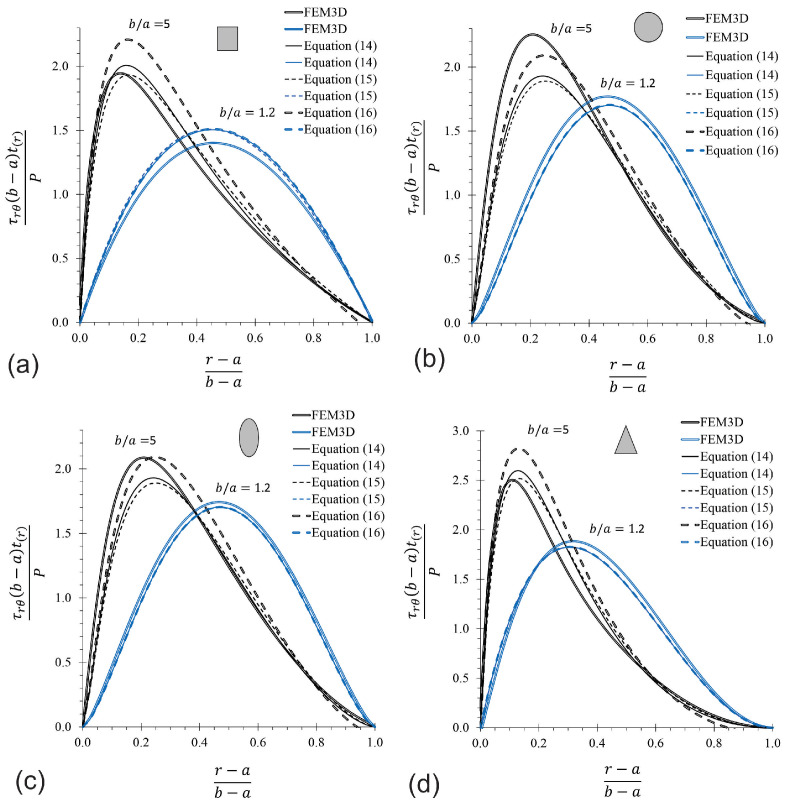
Comparison between FEM and MM solutions for CBs with ratios of b/a= 5 and 1.2, and SB: (**a**) rectangular, (**b**) circular, (**c**) elliptical, and (**d**) triangular cross-section (For midplane axis at θ=0°).

**Figure 9:**
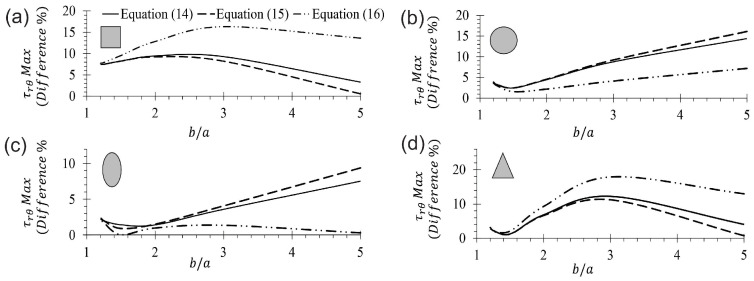
Relative difference in maximum $\tau_{r\theta }$ predictions between MM solutions and FEM. (**a**) Rectangular, (**b**) circular, (**c**) elliptical, (**d**) triangular cross-section.

The maximum shear stress ratio obtained for a CB using Equation (14) and FEM for rectangular, circular, elliptical, and triangular cross-sections are listed in Table 3. The difference between the elasticity solution and FEM is less than 5.5% for the CB with a rectangular cross-section, and is attributed to the influence of the Poisson’s ratio, which is considered in 3D FEM simulations. The maximum error committed with respect to FEM when Equation (14) is used for predicting the shear stress is 14%, corresponding to CBs with a circular cross-section and ratios of b/a= 5. For the same section of CBs, but with ratios of b/a≤3, the error decreases to approximately 9%. However, the error is less than 4% for the other three sections when b/a≤3. The agreement among the current results suggests that Equation (14) is suitable for predicting the shear stress $\tau_{r\theta }$ in a CB with ratios of b/a≤5. To explore the differences found, more rigorous studies of the MM solutions (Equations (14)–(16)) are presented in Figure 8 and Figure 9, respectively. Figure 8 compares FEM and MM solutions for CBs with ratios of b/a= 5 and 1.2 for the four considered cross-sections. This figure shows differences among MM equations and FEM predictions, especially for ratios of b/a= 5, and these differences are strongly reduced as b/a tends to unity. Figure 9a corresponds to a rectangular cross-section, and Figure 9d corresponds to a triangular cross-section. These figures showed a difference of approximately 10%, with Equation (15) being slightly more accurate than Equation (14), while the difference with Equation (16) is more appreciable (around 16%). Curiously, this trend is opposed to the circular and elliptical cross-sections represented in Figure 9b,c, respectively. Equation (14) has a maximum difference of 16.0%, being slightly more precise than Equation (15), but Equation (16) shows better results, with a maximum difference of 7.5% for elliptical cross-section with b/a= 5, while for circular cross-section, this is reduced to a maximum of 1%, which is practically uniform for the whole analyzed interval (1.2≤b/a≤5). These findings obtained across Figure 8 and Figure 9 indicate that the corrections made by Cook [36] to predict the circumferential stress are a good approximation for predicting the shear stress on CBs, and are close to the exact solution. Particularly, of the three equations, it is preferred to use Equation (14) because, in general, its predictions lie between those of the other two equations. Nevertheless, Equation (15) is simpler than the other two.

Table 4 shows results for a CB with a rectangular cross-section with normalized radii (r−a)/(b−a) and b/a ratios of 5 and 3, obtained from the Timoshenko [26], Oden [40], Yu and Nie [42], and Liu [41] solutions, as well as from current Equations (14)–(16) developed herein. The results from Yu’s model are consistent with those predicted by Oden’s model, and are more accurate than those from Liu’s model in relation to the Timoshenko solution. However, they are still significantly less accurate compared to the current solutions with respect to the elasticity solution.

### 3.5. 3D Shear Stress Profiles from FEM

**Figure 10:**
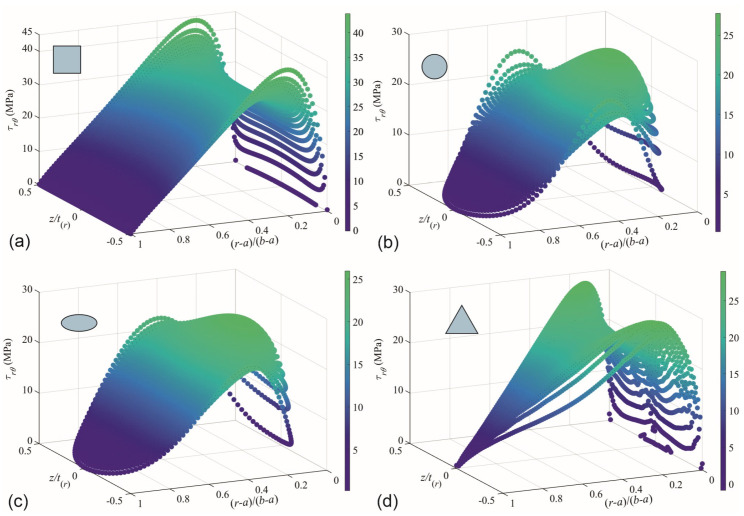
FEM−3D profile of CBs with ratio of b/a=3. (**a**) Rectangular, (**b**) circular, (**c**) elliptical, and (**d**) triangular cross-section (for midplane axis at θ=0°).

The stress field of $\tau_{r\theta }$ at the midplane of the cross-section in θ=0° for CBs with a ratio of b/a= 3 were obtained by FEM. Figure 10 shows the stress field for rectangular, circular, elliptical, and triangular cross-sections. Figure 10 shows a $z/t_{\left(r\right)}$ axis, which represents the normalized coordinate *z* respect to the thickness $\left(-0.5\leq z/t_{\left(r\right)}\leq 0.5\right)$, and a shear stress $\tau_{r\theta }$ reached by the CB when a uniform displacement of δ=0.1 mm is induced, as shown in Figure 3b. Upon examining the four charts, it is noticed that in the CBs with rectangular (Figure 10a) and triangular (Figure 10d) cross-sections, $\tau_{r\theta }$ reaches maximum peaks of 43.8 MPa and 29.0 MPa, respectively, at the edges $\left(z/t_{\left(r\right)}=\pm 0.5\right)$ of the neutral axis ((r−a)/(b−a)= 0.238 and 0.175). The difference in $\tau_{r\theta }$ estimation between the edges and the center for these cases, in the same order as discussed, is approximately 29% and 20%, respectively. On the other hand, the maximum $\tau_{r\theta }$, in the CBs with circular (Figure 10b) and elliptical (Figure 10c) cross-sections is reached at the center $\left(t/t_{\left(r\right)}=0\right)$ of the neutral axis ((r−a)/(b−a)= 0.275 and 0.300), with values of 27.8 MPa and 26.0 MPa, respectively. The difference in $\tau_{r\theta }$ estimation between the edges and the center for these latter two cases, in the same order discussed, is approximately 16% and 23%. Also, it was noticed that the $\tau_{r\theta }$ is not uniform on the whole cross-section, and the variation is more remarkable on the neutral axis. It is important to mention that this variation along the thickness is reduced when the b/a ratio diminishes to 1.5 (verified, but not shown here). These observations allow us to conclude that if the prediction of $\tau_{r\theta }$ is made at the ends of midplane for CBs with ratios of b/a≤2, the MM solutions can be reliably applied with an error less than 8%, for ratios of 2<b/a≤5.

## 4. Conclusions

In this study, three distinct equations for shear stress $\tau_{r\theta }$ in curved beams were derived using a mechanics of materials approach. These equations were validated against finite element method (FEM) simulations and theoretical solutions for rectangular cross-sections. The validation revealed the following key findings:

- The proposed equations demonstrated an overall accuracy within approximately 16%, with an improved accuracy of less than 8% for curved beams where 2<b/a≤5 and b/a≤2, respectively.
- The neutral axis location was predicted with high accuracy for beam ratios 1≤b/a≤5, with an absolute deviation of 0.03·(r−a)/(b−a).
- Near the edges of the curved beams, FEM analysis indicated that both the mechanics of materials equations and the theory of elasticity solutions (for rectangular cross-sections) provide a shear stress prediction error of approximately 29% for 2<b/a≤5. However, for ratios b/a≤2, the error decreases significantly, and any of the three mechanics of materials solutions can be applied with a small error of less than 8%.
- For structural solids with ratios b/a>5, it is recommended to use numerical simulations via FEM for accurate prediction of the shear stress field.
- The current equations can be reduced to Collignon’s formula, which proves to be a reliable option for predicting shear stress in curved beams with ratios b/a≤1.2.
- The current solutions do not apply to CB structures with thin flanges (such as I-, L-, or T-shaped cross-sections), as they produce significant errors. These errors arise from discontinuities in the cross-section, affecting tangential stress distribution. Since the current solutions rely on accurate tangential stress calculations, they are directly impacted by this issue. In such cases, a correction factor must be developed or assessed whether Bleich’s methods can be applied.

In summary, while the proposed equations offer reasonable accuracy across a range of beam geometries, FEM simulations are advised for cases with extreme aspect ratios. Collignon’s formula remains a valuable tool for beams with small b/a ratios.

## Figures and Tables

**Table 1:** Dimensions of the CBs analyzed keeping b−a= 100 (mm).

*b/a*	*a*	*b*	(*a* + *b*)/2
5	20	100	60
3	50	150	100
2	100	200	150
1.5	200	300	250
1.25	400	500	450
1.2	500	600	550

where (b−a) is the depth of the beam.

**Table 2:** FEM predictions of neutral axis location for CB.

CB		(r−a)/(b−a)		
*b/a*	Rectangular	Circular	Elliptical	Triangular
5.00	0.138	0.200	0.200	0.100
3.00	0.238 (0.244 *)	0.275	0.300	0.175
2.00	0.338 (0.331 *)	0.350	0.375	0.250
1.50	0.400	0.400	0.425	0.300
1.25	0.450	0.450	0.450	0.325
1.20	0.450	0.475	0.475	0.325
SB	0.500	0.500	0.500	0.333

*: 2D theory of elasticity solution [26].

**Table 3:** Maximum shear stress ratio between Equation (14) and FEM at θ=0° $\left(\tau_{r\theta }^{14}/\tau_{r\theta }^{FEM}\right)$.

CB		Cross-Section		
*b/a*	Rectangular	Circular	Elliptical	Triangular
5.00	1.03	0.86	0.92	1.04
3.00	1.09 (1.06 *)	0.91	0.96	1.12
2.00	1.09 (1.03 *)	0.96	0.99	1.07
1.50	1.08	0.98	0.99	1.01
1.25	1.07	0.97	0.98	0.98
1.20	1.07	0.96	0.98	0.97

*: 2D theory of elasticity solution [26].

**Table 4:** Comparison among different solutions and current Equations (14)–(16) for shear stress in CB $\left(\tau_{r\theta }\left(b-a\right)t_{\left(r\right)}/P\right)$ with rectangular cross-section and ratios of b/a=5 and 3 (see, Table 1).

b/a	$\frac{\left(r-a\right)}{\left(b-a\right)}$	Elasticity	MM	MM	MM	MM	MM
		Ref. [26]	Refs. [40,42]	Ref. [41]	Equation (14)	Equation (15)	Equation (16)
5	0.00	0.000	0.000	0.000	0.000	0.000	0.000
	0.25	1.765	2.608	3.372	1.848	1.811	1.782
	0.50	1.063	1.500	2.249	1.038	1.073	1.023
	0.75	0.473	0.633	1.112	0.414	0.453	0.390
	1.00	0.000	0.000	0.318	0.000	0.000	−0.071
3	0.00	0.000	0.000	0.000	0.000	0.000	0.000
	0.25	1.674	1.990	2.248	1.707	1.690	1.817
	0.50	1.256	1.500	1.818	1.258	1.268	1.348
	0.75	0.619	0.720	0.921	0.593	0.608	0.625
	1.00	0.000	0.000	0.122	0.000	0.000	−0.040

**Figure A1:**
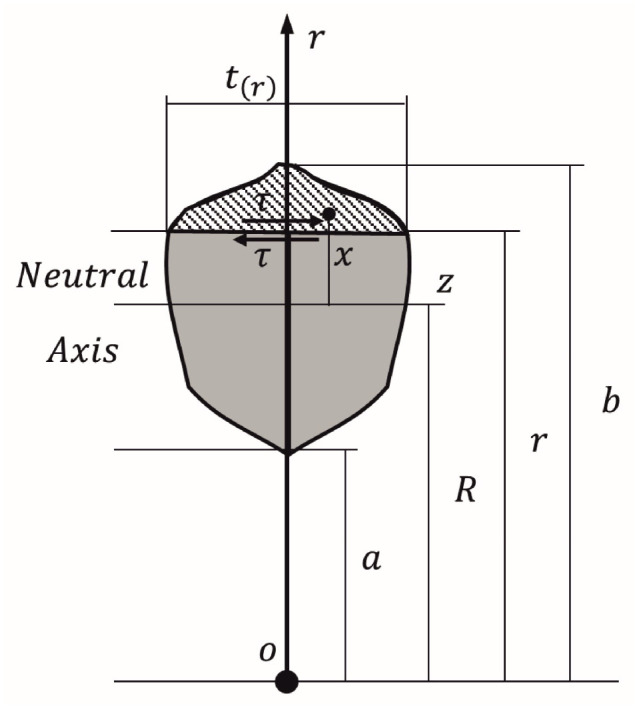
Cross-section for a CB with R≫x, such as x/R→00.

## Data Availability

The original contributions presented in the study are included in the article, further inquiries can be directed to the corresponding authors.

## Appendix A

### Appendix A.1. Reduction for Shear Stress Equation in Curved Beams to Collignon’s Formula for Straight Beams

Figure A1 shows the cross-section of a CB, which allows us to show the reduction of Equation (14) to Collignon’s formula. The border between the dashed and gray zone represents the line where the shear stress is calculated. Distance y is measured from the neutral axis of the CB to the differential element of study, and it is well known that, for CBs with R>>(b−a), this neutral axis coincides to the centroid, which agrees with the SB theory [36].

(A1)
$$\tau_{r\theta }=\left(\frac{A^{\prime }}{A_{m}}+\frac{\left(A_{m}R-A\right)\left(I^{\prime }-RQ\right)}{I_{m}A_{m}}+\frac{R(AA^{\prime }-A_{m}Q)}{A(RA_{m}-A)}\right)\frac{P\cos(\theta )}{r^{2}t}$$

The term Pcos(θ) is identified as the shear load (V) acting on the cross-section of the CB. Additionally, the first two terms in Equation (A1) are related to the normal load (N), and for SBs N=0. Thus, Equation (A1) is reduced to

(A2) $$\tau_{r\theta }=\left(\frac{R(AA^{\prime }-A_{m}Q)}{Ar(RA_{m}-A)}\right)\frac{V}{rt}$$

Boresi and Schmidt [61] showed that $r(RA_{m}-A)$ is reduced to $I_{z}/R$ when *R* is very large with respect to *y* (Figure A1). Thus, Equation (A2) is rewritten as

(A3) $$\tau_{r\theta }=\left(\frac{R^{2}\left(AA^{\prime }-A_{m}Q\right)}{AI_{z}}\right)\frac{V}{rt}$$

where $I_{z}$ is the second moment of area, and R is the distance from the centroid of the cross-section to the center of curvature. By separating the terms of the last equation and using the integral form of the terms $A^{\prime }$ and $A_{m}$, Equation (A3) is rewritten as

(A4) $$\tau_{r\theta }=\left(\frac{R^{2}}{r}\int_{a}^{r}dA-\frac{R^{2}Q}{Ar}\int_{a}^{b}\frac{dA}{r}\right)\frac{V}{tI_{z}}$$

From Figure A1, distance *r* is given by r=R+x. Now, replacing *r* by its new expression into Equation (A4) and rearranging

(A5) $$\tau_{r\theta }=\left(\frac{1}{1+\frac{x}{R^{2}}}\int_{a}^{r}dA-\frac{Q}{A\left(1+\frac{x}{R}\right)}\int_{a}^{b}\frac{dA}{1+\frac{x}{R}}\right)\frac{V}{tI_{z}}$$

Finally, taking the limit of Equation (A5) when *R* is too large with respect to *x*, the terms $x/R^{2}$ and x/R tend to vanish, and the shear stress equation for SBs, well-known as Collignon’s formula (A6), is obtained:

(A6) $$\tau_{r\theta }=-\frac{VQ}{tI_{z}}$$

where $Q_{\left(z\right)}$ is the first moment of area. The negative sign in Equation (A6) is due to the sign convention used on CBs. Equations (15) and (16) are also reduced to Equation (A6).
